# Short wavelength automated perimetry and standard automated perimetry in central serous chorioretinopathy

**DOI:** 10.1038/s41598-020-73569-0

**Published:** 2020-10-05

**Authors:** Han Peng Zhou, Ryo Asaoka, Tatsuya Inoue, Shotaro Asano, Hiroshi Murata, Takumi Hara, So Makino, Kazuaki Kadonosono, Ryo Obata

**Affiliations:** 1 Department of Ophthalmology, The University of Tokyo Hospital, 7-3-1 Hongo, Bunkyo-ku, Tokyo, 113-8655 Japan; 2 Department of Ophthalmology and Micro-Technology, Yokohama City University School of Medicine, 4-57 Urafune, Minami-ku, Yokohama, 232-0024 Japan

**Keywords:** Eye diseases, Retinal diseases, Vision disorders

## Abstract

Short wavelength automated perimetry (SWAP) is known for detecting the early reduction of retinal sensitivity (RS) in glaucoma. It’s application in retinal diseases have also been discussed previously. We investigated the difference in RS measured between standard white-on-white automated perimetry (WW) and blue-on-yellow SWAP in central serous chorioretinopathy (CSC). The overall RS (W-RS, S-RS) as well as the RS inside and outside of the serous retinal detachment (SRD) region were investigated in 26 eyes of 26 CSC patients using WW and SWAP. The central retinal thickness, central choroidal thickness, SRD area (SRDa), and SRD height at the fovea were measured using optic coherence tomography. RS inside the SRD region was lower than that of outside for both perimetries (both p < 0.001). The difference between RS inside and outside of the SRD region was greater in SWAP compared to WW (p < 0.001). Univariate analysis revealed significant correlations between SRDa and both W-RS and S-RS (both p < 0.001); moreover, multivariate analysis indicated that only S-RS was selected as the optimal model for SRDa. Our study demonstrated that SWAP was detected the decrease in RS more accurately than WW in CSC. These results may suggest the usefulness of SWAP for detecting change of retinal function in CSC.

## Introduction

Central serous chorioretinopathy (CSC) is a disease involving the posterior pole of the eye characterized by localized serous detachment of the neurosensory retina^1–3^. The exact etiology of CSC has yet to be revealed, although the use of glucocorticoids has been said to be involved in the increased permeability of the underlying choriocapillaris, causing the weakening of the retinal pigment epithelium and resulting in pigment epithelium detachment as well as serous retinal detachment (SRD)^4–8^. Other associations include pregnancy, stress, and type A personality^9^.

At the clinical settings, functional assessment of CSC is usually carried out using visual acuity (VA). Most initial retinal detachments in CSC resolve spontaneously^10,11^; however, chronic CSC can also be experienced where patients often complain of visual symptoms, such as decreased visual sensitivity, metamorphopsia, and central scotoma^12–14^, despite the preserved VA. Agreeing with this, we have recently shown that it was advantageous to assess the visual function in chronic CSC using the white-on-white (WW) visual field (VF) testing (with microperimetry) rather than using VA alone^15^. The retinal sensitivities, but not VA, were well associated with SRD measurements obtained by optical coherence tomography (OCT)^15^.

Short wavelength automated perimetry (SWAP), or blue-on-yellow perimetry, selectively evaluates the function of short-wavelength-sensitive (SWS), or blue cone, pathway by projecting a blue stimulus on a yellow background^16^. SWAP is known for detecting reduction of retinal sensitivity (RS) in advance of standard automatic perimetry in glaucoma due to the sparse distribution of retinal ganglion cells which mediates blue stimuli^10–14,17–22^, although more recent studies have revealed the limited usefulness of this approach^23,24^. Similarly, the damaged function of macular photoreceptor cells (even with preserved VA) in CSC may be more sensitively detected with SWAP than with WW, due to the sparse distribution of retinal photoreceptor cells which medicate blue stimuli^25^. Nonetheless, there is only limited number of studies which has shed light on this^25^ and detailed examination has not been sufficiently performed.

In the current study, we evaluated visual function using SWAP and WW perimetries in CSC patients with SRD, and compared the usefulness in relation to OCT-measured structure of SRD.

## Results

The characteristics of the study subjects are summarized in Table 1. The mean age of the participants was 55.3 ± 11.5 [34 to 76] (mean ± standard deviation [range]) years and the mean logMAR best-corrected visual acuity (BCVA) was 0.053 ± 0.15 [− 0.079 to 0.52]. The mean overall RSs of WW (W-RS) was 30.0 ± 2.3 [22.3 to 32.7]. The mean RS inside and outside the SRD-affected regions in WW (W-RS_in and W-RS_out, respectively) was 29.3 ± 2.9 [21.0 to 34.3] and 30.6 ± 2.0 [23.4 to 32.9] dB, respectively. Meanwhile, the mean overall RSs of SWAP (S-RS) was 25.6 ± 3.5 [18.1 to 34.2]. The mean RS inside and outside the SRD-affected regions in SWAP (S-RS_in and S-RS_out, respectively) was 23.0 ± 3.3 [16.2 to 30.0] and 26.6 ± 2.9 [19.7 to 31.5] dB, respectively. Central retinal thickness (CRT) and central choroidal thickness (CCT) was 164.5 ± 42.8 [111.0 to 280.0] and 417 ± 124.1 [218.0 to 672.0] µm, respectively. SRD height at the fovea (SRDh) and the area sum of all observed SRD regions (SRDa) was 117.8 ± 83.5 [0.0 to 264.0] µm and 6.43 ± 5.3 [0.13 to 20.24] mm^2^, respectively.

**Table 1:** Subject demographics.

	Mean ± SD (n = 26)	Range
Age (years)	55.3 ± 11.5	34 to 76
Gender ratio (male:female)	20:6	–
Eye (right:left)	7:19	–
WW test duration (seconds)	442.6 ± 105.7	329 to 659
SWAP test duration (seconds)	430.9 ± 86.4	305 to 666
LogMAR BCVA	0.053 ± 0.153	− 0.079 to 0.523
W-RS (dB)	30.0 ± 2.3	22.3 to 32.7
W-RS_in (dB)	29.3 ± 2.9	21.0 to 34.3
W-RS_out (dB)	30.6 ± 2.0	23.4 to 32.9
S-RS (dB)	25.6 ± 3.49	18.1 to 34.2
S-RS_in (dB)	23.0 ± 3.3	16.2 to 30.0
S-RS_out (dB)	26.6 ± 2.9	19.7 to 31.5
SRDa (mm^2^)	6.43 ± 5.3	0.13 to 20.24
SRDh (µm)	117.8 ± 83.5	0.0 to 264.0
CRT (µm)	164.5 ± 42.8	111.0 to 280.0
CCT (µm)	417.0 ± 124.1	218.0 to 672.0

*WW* white-on-white automated perimetry, *SWAP* short wavelength automated perimetry, *logMAR BCVA* logarithm of the minimum angle of resolution of best-corrected visual acuity, *SRD* serous retinal detachment, *W-RS* mean overall RS of WW, *W-RS_in* mean RS inside the SRD regions in WW, *W-RS_out* mean RS outside the SRD regions in WW, *S-RS* mean overall RS of SWAP, *S-RS_in* mean RS inside the SRD regions in SWAP, *S-RS_out* mean RS outside the SRD regions in SWAP, *SRDa* area sum of all observed SRD regions (foveal and extra-foveal), *SRDh* SRD height at the fovea, *CRT* central retinal thickness, *CCT* central choroidal thickness.

**Figure 1:**
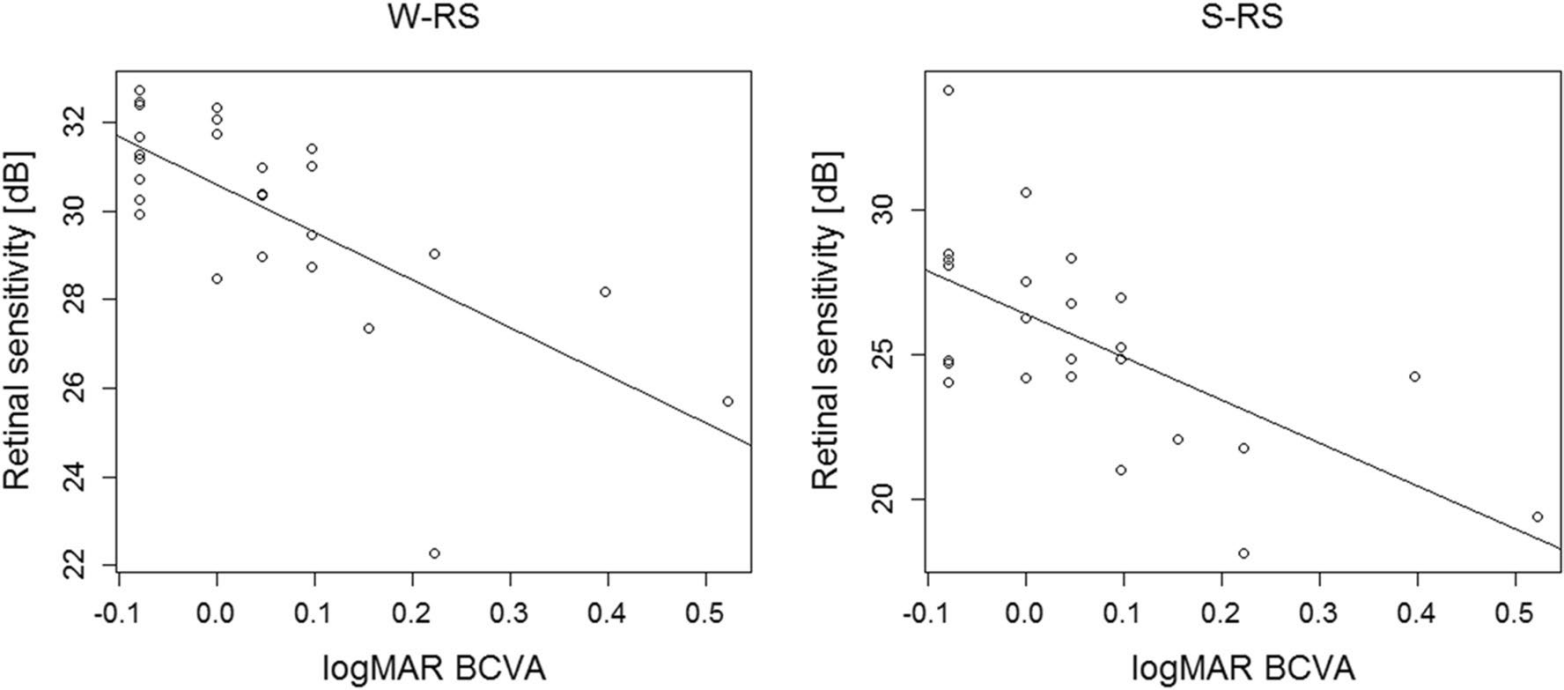
Correlations between logMAR best-corrected visual acuity (BCVA) and retinal sensitivity. Significant negative correlations were observed between logMAR BCVA and the overall mean retinal sensitivity of white-on-white perimetry (W-RS) as well as short wavelength automated perimetry (S-RS) (p = 6.44 × 10^−5^ and 0.000313, respectively, linear regression).

**Figure 2:**
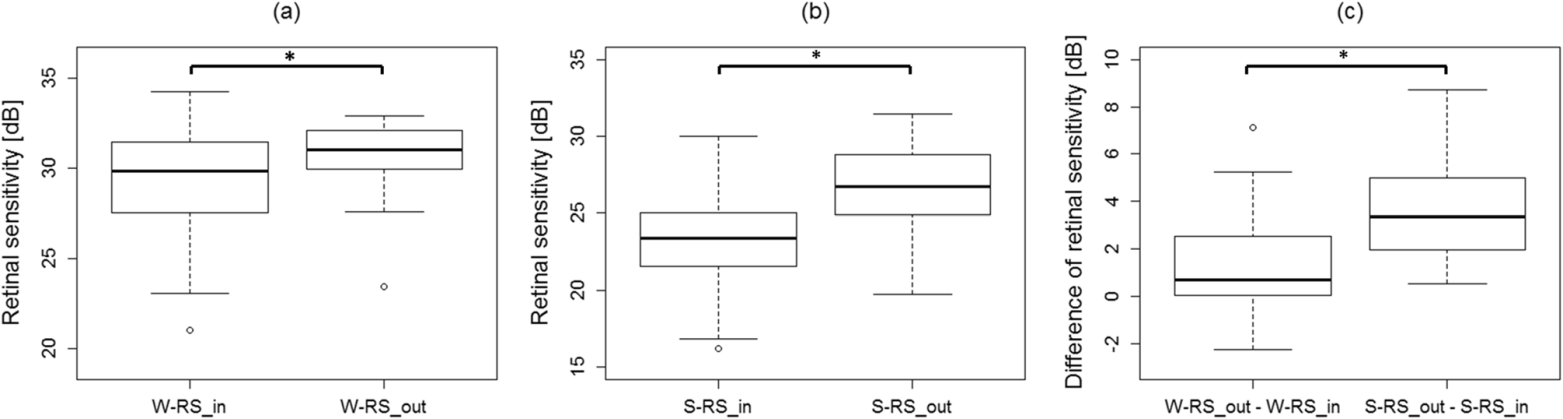
Correlations between retinal structure and retinal sensitivity. (**a**,**b**) Retinal sensitivity inside the serous retinal detachment region (W-RS_in, S-RS_in) was significantly lower than the outside (W-RS_out, S-RS_out) in both white-on-white (WW) perimetry (p = 0.006) and short wavelength automated perimetry (SWAP) (p = 0.006 and p = 2.98 × 10^−8^, exact Wilcoxon signed rank test). (**c**) The difference between the retinal sensitivity inside and outside of the SRD-affected regions was significantly larger in SWAP compared to WW (p = 5.66 × 10^−7^, exact Wilcoxon signed rank test).

Significant negative correlations were observed between logMAR BCVA and W-RS as well as S-RS (p = 6.44 × 10^−5^ and 0.000313, respectively, linear regression) (Fig. 1). W-RS_in and S-RS_in was significantly lower than W-RS_out and S-RS_out, respectively (p = 0.006 and p = 2.98 × 10^−8^, exact Wilcoxon signed rank test) (Fig. 2a,b).

Furthermore, the difference between S-RS_in and S-RS_out (3.7 ± 2.3 [0.5 to 8.7] dB) was significantly larger than that of W-RS_in and W-RS_out (1.3 ± 2.2 [− 2.2 to 7.1]) dB) (p = 5.66 × 10^−7^, exact Wilcoxon signed rank test) (Fig. 2c).

The results of univariate analysis are shown in Tables 2, 3, 4 and 5. LogMAR BCVA was significantly correlated to age, CCT, and SRDa (coefficient = 0.0063, − 0.00055, and 0.0053, respectively, and p = 0.015, 0.023, and 0.025, respectively, linear regression). W-RS was significantly correlated to age and SRDa (coefficient =  − 0.097 and − 0.28, respectively, and p = 0.014 and 0.001, respectively). S-RS was also significantly correlated to age and SRDa (coefficient =  − 0.17 and − 0.44, respectively, and p = 0.003 and p = 0.00024, respectively). Both W-RS and S-RS negatively correlated with SRDa (coefficient =  − 1.44 and − 1.003, respectively, and p = 0.00048 and 0.00024, respectively).

**Table 2:** Result of univariate analysis between logMAR best-corrected visual acuity (BCVA) and optical coherence tomography parameters.

Variables	Coefficient of correlation	Coefficient	Standard error	p-value
Age	0.47	0.0063	0.0024	0.015
CRT	− 0.16	− 0.00057	0.00073	0.44
CCT	− 0.44	− 0.00055	0.00023	0.023
SRDa	0.44	0.013	0.0053	0.025
SRDh	− 0.008	− 0.000015	0.00037	0.97

*CRT* central retinal thickness, *CCT* central choroidal thickness, *SRDa* area sum of all observed SRD regions (foveal and extra-foveal), *SRDh* SRD height at the fovea.

**Table 3:** Result of univariate analysis between the mean overall retinal sensitivity of white-on-white automated perimetry (W-RS) and optic coherence tomography parameters.

Variables	Coefficient of correlation	Coefficient	Standard error	p-value
Age	− 0.48	− 0.097	0.037	0.014
CRT	0.24	0.014	0.011	0.23
CCT	0.25	0.0048	0.0037	0.21
SRDa	− 0.64	− 0.28	0.070	0.001
SRDh	− 0.33	− 0.0092	0.0054	0.102

*CRT* central retinal thickness, *CCT* central choroidal thickness, *SRDa* area sum of all observed SRD regions (foveal and extra-foveal), *SRDh* SRD height at the fovea.

**Table 4:** Result of univariate analysis between the mean overall retinal sensitivity of short wavelength automated perimetry (S-RS) and OCT parameters.

Variables	Coefficient of correlation	Coefficient	Standard error	p-value
Age	− 0.56	− 0.17	0.052	0.003
CRT	0.13	0.011	0.017	0.53
CCT	0.16	0.0046	0.0057	0.43
SRDa	− 0.66	− 0.44	0.1009	0.00024
SRDh	− 0.31	− 0.013	0.0081	0.13

*CRT* central retinal thickness, *CCT* central choroidal thickness, *SRDa* area sum of all observed SRD regions (foveal and extra-foveal), *SRDh* SRD height at the fovea.

**Table 5:** Result of univariate analysis between the total SRD area (SRDa) and retinal sensitivities.

Variables	Coefficient of correlation	Coefficient	Standard error	p-value
logMAR BCVA	0.44	15.25	6.36	0.0247
W-RS	− 0.64	− 1.44	0.36	0.00048
S-RS	− 0.66	− 1.003	0.23	0.00024

*logMAR BCVA* logarithm of minimum angle of resolution of best-corrected visual acuity, *W-RS* mean overall retinal sensitivity of white-on-white automated perimetry, *S-RS* mean overall retinal sensitivity of short wavelength automated perimetry.

The optimal models for logMAR BCVA, W-RS, and S-RS, as a result of multivariate analysis using Akaike’s information criterion (AIC) model selection, are shown below:

- logMAR BCVA = 0.257 − 0.00059 × CCT (standard error: SE = 0.00019, p = 0.0064) + 0.016 × SRDa (SE = 0.016, p = 0.0036) − 0.00054 × SRDh (SE =  − 0.00054, p = 0.105) (AICc =  − 27.9).
- W-RS = 36.469 − 0.086 × Age (SE =  − 0.086, p = 0.0054) − 0.26 × SRDa (SE =  − 0.26, p = 0.00022). (AICc = 104.6)
- S-RS = 36.617 − 0.15 × Age (SE = 0.036, p = 0.00031) − 0.40 × SRDa (SE = 0.078, p = 2.92 × 10^−5^) (AICc = 117.8).

The optimal model for SRDa, as a result of multivariate analysis using AIC model selection, is shown below:

- SRDa = 32.128 − 1.003 × S-RS (SE = 0.23, p = 0.00024) (AICc = 151.7).

## Discussion

In the present study, RS was measured using SWAP and WW perimetric tests in 26 CSC eyes with SRD, along with the SRD measurements with OCT. As a result, it was suggested that the difference of the retinal sensitivities within and outside the SRD region was significantly larger with SWAP than with WW perimetry. Furthermore, the optimal model for SRDa only included S-RS but not W-RS. These outcomes suggest the usefulness of SWAP in the evaluation of visual functions in CSC eyes compared to WW perimetry.

Previous studies have shown the usefulness of SWAP in evaluating retinal functions in some retinal diseases such as diabetic retinopathy^26–39^, age-related macular degeneration^40–43^, birdshot retinochoridopathy^44,45^, inherited retinal degeneration^46^, idiopathic optic neuropathy^47^, Afrashi et al. has investigated the change in the central RS in 18 resolved CSC patients and compared to that in normal controls, using SWAP and WW perimetries^25^. As a result, the mean deviation value was significantly lower in the CSC group compared with the control group for both SWAP and WW perimetry, despite the preserved VA (20/20) in all subjects with CSC. With SWAP, other perimetric parameters (pattern standard deviation, short-term fluctuation, and corrected pattern standard deviation) were also significantly lower in the CSC group than in the control group, which was not the case with WW perimetry. This result suggests the potential usefulness of SWAP in eyes with CSC over WW perimetry; however, much more detailed investigation is required, as this study merely compared the overall RSs in the measured VF. In contrast, the current study investigated RSs inside and outside the SRD-affected region separately both with SWAP and WW perimetries. In general, RS decreases as the eccentricity from the fovea increases^48,49^. Nonetheless, in the current study, W-RS_in was significantly lower than W-RS_out agreeing with previous studies (Fig. 2a)^15,50^. Similar tendency was observed in the comparison between S-RS_in and S-RS_out (Fig. 2b). It should be postulated that the difference between S-RS_in and S-RS_out was significantly larger than between W-RS_in and W-RS_out (Fig. 2c). This cannot be fully explained by the difference of W-RS and S-RS, since S-RS was significantly lower than W-RS. Indeed an additional experiment using the difference rate relative to W-RS and S-RS yielded a significant large difference rate with SWAP (data not shown in Result).

In our previous study, we investigated the association between macular volume of SRD and the RS at 0, 2, 4, and 6 degrees from the fovea, which suggested that RS was significantly associated with macular volume at the corresponding region, and not at other regions^15^. LogMAR BCVA mainly reflects the retinal function around the fovea only, whereas RS is the result of the retinal function in a wider area. Agreeing with this, in the current study, SRDh was included in the optimal model for logMAR BCVA but not in those for W-RS and S-RS. In contrast to SRDh, SRDa is a structural parameter obtained from a wider region of the retina, and indeed, SRDa was included in the optimal models for both W-RS and S-RS. This is in agreement with a previous study by Sekine et al. in that a significant decrease of RS was observed in the SRD-affected region^52^. Of note, it should be postulated that W-RS was not included in this optimal model for SRDa, which suggests that SWAP was more useful than WW perimetry when analyzing the structure–function relationship using SRDa.

SWAP selectively evaluates the function the blue cone pathway by projecting a blue stimulus on a yellow background, which hyperpolarizes the rods as well as other long- and middle-wavelength-sensitive cones^16,51,52^. Only about 5–10% of the cone cells in the retina are blue cones^10–14,17–22,51^. In particular, they are distributed less in number around the fovea compared to the surrounding retina^48^. In glaucoma, this sparse distribution of blue cone pathways has been reported to enable early detection of VF damage^13,18^. For instance, Johnson et al. and Sample et al. have reported that SWAP deficits are predictive of impending glaucomatous VF loss for standard WW perimetry in their longitudinal studies using ocular hypertensive and glaucomatous eyes^13,18^. Additionally, Machida et al. suggested that blue cone pathways may be more delicate and susceptible to photoreceptor diseases^53^. The response range of these cones is also significantly limited compared with the other cones, hence an equivalent loss of function among the cone cells would result in a larger response in the blue cone pathway^53^.

A limitation of the current study was that it was not possible to measure the foveal sensitivity using SWAP. Such measurement will enable further detailed investigation of the association with SRDh and visual function with SWAP and WW perimetry. Moreover, it may be possible for some regions of the retina outside of the SRD region to have locally decreased RS as they may indicate areas of resolved SRD from the past, retinal atrophy, or retinal pigment epithelium cracks. Since this was a cross-sectional study, we did not follow up on the disease duration and clinical course of each subject. It is of our interest to further investigate using image tools, such as fundus autofluorescence, to highlight such atrophic regions in areas outside of the current SRD and assess their retinal sensitivity difference.

In conclusion, our study demonstrated that the difference between RS outside and inside of the SRD region was greater in SWAP than WW, suggesting that SWAP is more sensitive in detecting retinal dysfunction in CSC eyes. Furthermore, SRDa was better correlated with W-RS than W-RS, suggesting that SWAP is more sensitive to morphological change in CSC eyes. These results suggest the usefulness of SWAP for evaluating the retinal function as a result of structural changes of the retina in CSC patients.

## Methods

This study was approved by the Research Ethics Committee of the Graduate School of Medicine and Faculty of Medicine at The University of Tokyo (Approval ID: 3770). Informed consent was obtained from all participating subjects. This study was performed according to the tenets of the Declaration of Helsinki.

Cross-sectional case series was employed in the current study. Twenty-six eyes of 26 CSC patients (20 males and 6 females) with intra and/or extra foveal SRDs who visited The University of Tokyo Hospital were included as subjects of this study. All patients underwent comprehensive ophthalmological examinations including measurement of BCVA and intra-ocular pressure, anterior segment examination, and fundoscopy under pupil dilation. The diagnosis of CSC was based on findings from OCT, fluorescein angiography, and indocyanine green angiography. All subjects underwent OCT imaging, as well as SWAP and WW perimetric tests on the same day. Eyes with other ocular diseases such as age-related macular degeneration and choroidal neovascularization were carefully excluded.

BCVA was measured using the Landolt chart and were converted to logMAR BCVA for analysis. Spectral domain OCT images were obtained using the Spectralis OCT (Heidelberg Engineering, Heidelberg, Germany). All OCT images consisted of line scans (horizontal and vertical B-scans) and raster scans (25 horizontal B-scans). CRT, CCT, and SRDh were measured using images obtained from enhanced depth imaging (EDI) mode. SRDa was measured by evaluating each of the 25 B-scans for SRD; the edges of the SRD were marked on each scan. All markers indicating the edge of the SRD were then connected to identify the SRD-affected area. If more than one isolated SRD region existed in the eye, SRDa was calculated as the area sum of all observed SRD regions.

Both SWAP and WW perimetric tests were performed using the AP-7000 automatic perimeter (KOWA Company Ltd., Tokyo, Japan) using the full-threshold strategy (Goldmann size III). RS thresholds were measured within 10-degree radius from the fovea, corresponding to the Humphrey Field Analyzer 10-2 program (Carl Zeiss Meditec, Dublin, CA). SWAP was conducted using a 450 nm blue narrow band target which was presented for 200 ms, similarly to the WW perimetric test. The target was projected on a yellow background (600 nm with a luminance of 100 cd/m^2^). The order of SWAP and WW was performed randomly in order to minimize the learning effect of perimetry. Fixation point was checked using MP-3 microperimetry (Nidek Co. Ltd., Aichi, Japan) in all subjects.

**Figure 3:**
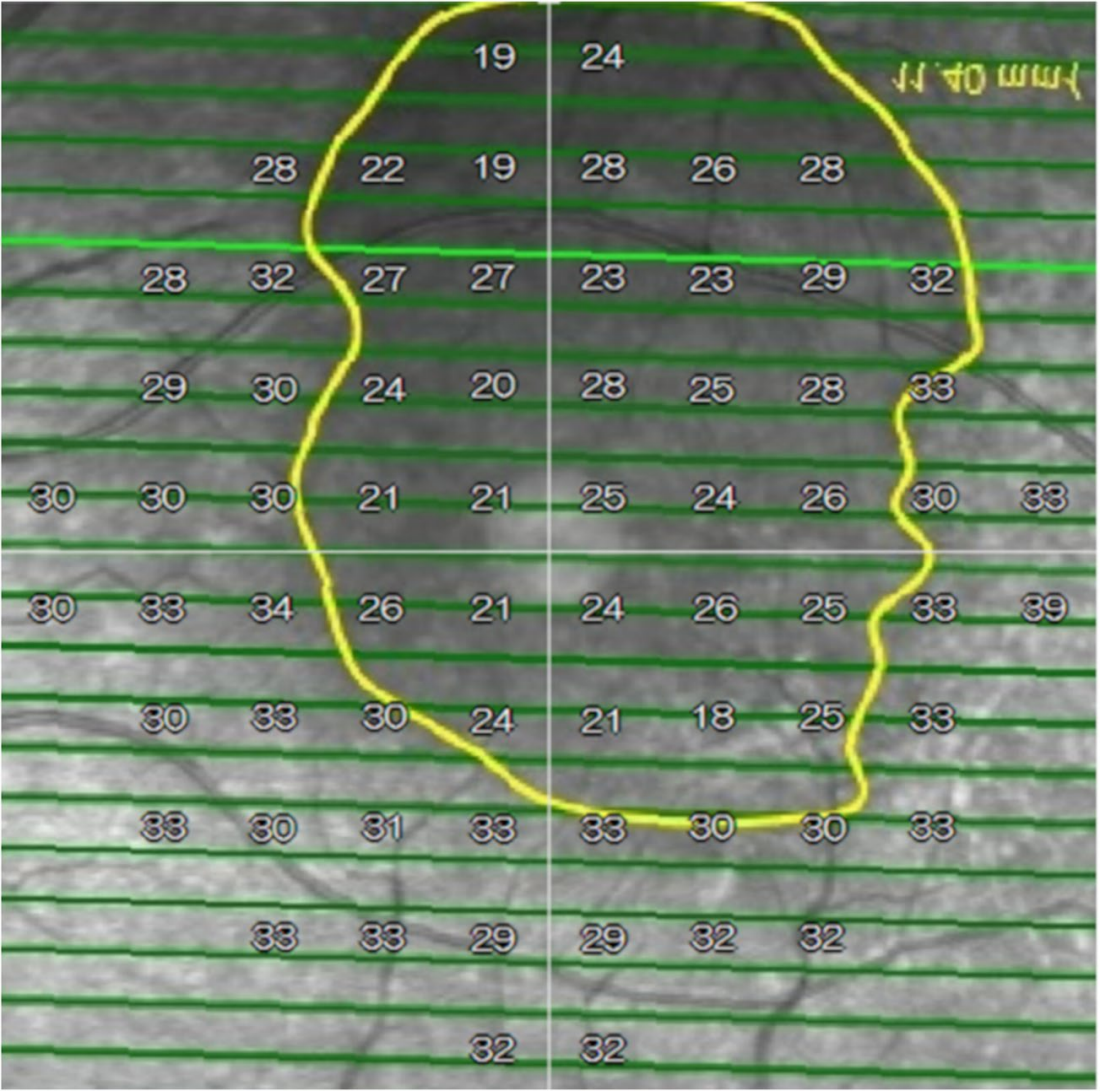
The optical coherence tomography image of the serous retinal detachment region and the perimetry results were superimposed using the built-in software in the AP-7000 automatic perimeter (AP-7000 Software Ver.3.0.6.0, KOWA Company Ltd., Tokyo, Japan). The software automatically inverts the retinal image to correspond to the visual field results. Hence, the image of the retina will remain inverted in the overlapped image, indicating that the images were correctly overlapped.

The SWAP and WW perimetry results were superimposed on the corresponding OCT images including the marked SRD region using the built-in software in the AP-7000 automatic perimeter (Fig. 3).

W-RS and S-RS were calculated. In addition, the mean RS inside (W-RS_in, S-RS_in) and outside (W-RS_out, S-RS_out) of the SRD-affected regions were also calculated.

### Statistical analysis

Comparisons were conducted (i) between W-RS_in and W-RS_out, (ii) between S-RS_in and S-RS_out, (iii) between W-RS_in and S-RS_in, and (iv) between W-RS_out and S-RS_out, using the exact Wilcoxon signed rank test. Subsequently, the differences between W-RS_in and W-RS_out and also between S-RS_in and S-RS_out were calculated and compared to each other using the exact Wilcoxon signed rank test.

Additionally, for each of the visual function parameters (logMAR BCVA, W-RS, and S-RS), the association with age and the four structural parameters (CCT, CRT, SRDh, and SRDa) was analyzed using univariate and multivariate linear regressions. Following the multivariate linear regression, model selection was performed to identify the optimal linear regression models using the second-order bias-corrected AICc index from all 2^5^ patterns consisting of five variables (age, CCT, CRT, SRDh, and SRDa). The AIC is a well-known statistical measurement used in model selection, and the AICc is a corrected version of AIC, which provides an accurate estimation even when the sample size is small^54,55^. The selected variables through the model selection were regarded as statistically significant. Similarly, the association between SRDa and visual function parameters (logMAR BCVA, W-RS, and S-RS) was analyzed and the optimal model was identified.

All statistical analysis was performed using the statistical programming language “R” (R version 3.5.1; The R Foundation for Statistical Computing, Vienna, Austria).

## Data Availability

The datasets generated and analyzed during the current study are available from the first/corresponding authors on reasonable request.
